# Plant compartment and understory habitat jointly shape endophytic bacterial communities in *Monochasma savatieri*

**DOI:** 10.3389/fmicb.2026.1930478

**Published:** 2026-10-02

**Authors:** Yuling Zou, Fengqing Li, Suzhen Liu, Wendi Lan, Mansheng Zeng, Guisheng Luo, Wei Huang, Lili Shen, Baolian Zhang, Shutong Sang

**Affiliations:** Experimental Center of Subtropical Forestry, Chinese Academy of Forestry, Xinyu, China

**Keywords:** community diversity, endophytic bacteria, functional prediction, *Monochasma savatieri*, understory habitat

## Abstract

**Introduction:**

*Monochasma savatieri* is a rare medicinal plant endemic to China that currently faces extinction, with wild-simulated understory cultivation representing a key conservation strategy. Endophytic bacteria play important roles in nutrient acquisition and environmental adaptation of hemiparasitic plants; however, how the understory environments influence above- and belowground endophytic bacterial communities remains poorly understood.

**Methods:**

We collected soil and plant tissue samples from four contrasting understory habitats (*Pinus massoniana*, *Camellia oleifera*, *Phyllostachys edulis*, and *Gardenia jasminoides*). The endophytic bacterial communities were characterized by 16S rRNA gene sequencing. The soil physicochemical properties were measured to identify environmental drivers. Multivariate statistics, including permutational multivariate analysis of variance (PERMANOVA), redundancy analysis (RDA), and Mantel tests, were applied to link the community variation with environmental factors.

**Results:**

Permutational multivariate analysis of variance revealed that the type of plant tissue, habitat, and their interaction (all *p* < 0.001) significantly shaped the endophytic community structure. Belowground tissues generally harbored higher diversity than aboveground tissues, although this pattern was habitat-dependent. Each habitat enriched distinct bacterial genera. Functional predictions showed a complementary pattern: aboveground communities were enriched in sensing-related pathways, whereas belowground communities showed higher abundances of degradation-associated pathways. RDA identified the soil total and available potassium as strong environmental correlates of community differentiation.

**Discussion:**

The findings of this study demonstrate that plant compartment and understory habitat jointly shape endophytic bacterial communities in *M. savatieri*, with potassium availability as a key correlate. However, because each habitat was represented by a single geographic site, the vegetation type is confounded with the location, climate, and soil properties; therefore, our inferences are correlative rather than causal. Within these constraints, the findings help identify candidate taxa and environmental associations that provide testable hypotheses for future cultivation and microecological research on this rare medicinal plant.

## 1 Introduction

*Monochasma savatieri* Franch. ex Maxim (Orobanchaceae) is a perennial hemiparasitic herb endemic to China, valued for its medicinal properties. In the wild, it is mainly distributed in low mountainous and hilly regions of eastern, southern, and central China (e.g., Jiangxi, Zhejiang, Fujian, and Hubei), typically at altitudes of 100–1200 m. It often occurs on sunny grassy slopes or under *Pinus massoniana* forests and shrublands, preferring a subtropical humid monsoon climate and acidic sandy loam soils (Chen et al., 2020b; Wu and Raven, 1998). Recent MaxEnt modeling predicts that its current highly suitable habitats are concentrated in Jiangxi, Zhejiang, Fujian, and adjacent provinces, with precipitation and temperature as the dominant climatic drivers (Luo et al., 2026). Its documented efficacy in heat-clearing, detoxification, pain alleviation, and blood-cooling for hemostasis underpins its role as a key ingredient in several nationally protected traditional Chinese medicinal formulations, underscoring its dual significance in pharmacotherapy and ecology (Zhang et al., 2024). Despite this importance, wild populations have declined dramatically due to persistent overharvesting and habitat degradation. This decline is exacerbated by extremely low seed germination rates under natural conditions, which severely restrict natural regeneration (Chen et al., 2020a). Consequently, understory cultivation that mimics natural growth conditions has emerged as an essential strategy for species conservation and sustainable utilization (Shi et al., 2022; Tang et al., 2022).

As a typical root hemiparasite, *M. savatieri* acquires water and mineral nutrients from host roots via specialized haustoria that establish xylem-to-xylem connections. Zhang et al. (2015) first reported its hemiparasitic nature and identified two moss species as primary hosts, along with 25 secondary host species. The establishment of parasitism significantly promotes growth, whereas growth remains extremely slow in the absence of hosts (Chen et al., 2020c). Notably, the intensity of this host-parasite interaction is habitat-dependent: under moderate anthropogenic disturbance, the relative population density can reach 64.1%, but it declines significantly in overly shaded or excessively disturbed habitats, with natural recruitment depending mainly on juvenile survival rates (Shi et al., 2022). Ecologically, hemiparasitic plants often act as “community regulators” by suppressing dominant hosts and altering competitive interactions (Tìšitel et al., 2021). This unique hemiparasitic lifestyle of *M. savatieri* likely profoundly influences its nutrient acquisition strategies and material exchange with the environment, thereby driving the specific assembly of its internal microbial communities. Yet, how understory habitat factors shape its endophytic bacterial communities–potentially through modulating host–parasite interactions–remains largely unexplored.

Despite previous investigations into its biological traits (Li et al., 2021), host selectivity (Zhang et al., 2015), soil requirements (Chen et al., 2020c), bioactive compound accumulation, and rhizosphere microbiota (Luo et al., 2024), a comprehensive understanding of the environmental mechanisms that govern the successful growth and establishment of *M. savatieri* remains lacking. Understory habitats are inherently complex, shaped collectively by vegetation type, microclimate, and soil properties (Chelli et al., 2024). These three dimensions do not operate in isolation; rather, climate, soil, and forest structure jointly drive understory community assembly and functional diversity (De Pauw et al., 2021). Nevertheless, the integrated ways in which these factors influence the ecophysiology of this species remain poorly elucidated.

Plant endophytes reside within plant tissues without causing overt disease and play central roles in the growth and adaptation of medicinal plants (Anand et al., 2023). These microbial communities support their hosts via nitrogen fixation, phosphate solubilization, and phytohormone secretion, while also modulating metabolism, synthesizing bioactive compounds, and enhancing stress tolerance (Trivedi et al., 2020). In hemiparasitic plants, endophytes may be particularly important drivers of adaptation to heterogeneous understory conditions, including mediating nutrient exchange between hosts and parasites (Adhikary et al., 2022; de Paula et al., 2022). The assembly of endophytic communities is a non-stochastic process and is subject to filtering by organ-specific microenvironments, aboveground conditions such as light, temperature, and humidity, and belowground factors such as soil type, pH, and nutrient content (de Paula et al., 2026). This selective process shapes distinct, host-adapted endophytic structures. Community function is closely linked to diversity, with consortia engaging in complex symbiotic, cooperative, and competitive relationships (Yu et al., 2019). Through processes such as quorum sensing, metabolite exchange, and horizontal gene transfer, endophytes form dynamic microecosystems that regulate host health, adaptability, and productivity (Kumar and Nautiyal, 2023; Oubohssaine et al., 2025). Accordingly, mechanistic insights into microbe–environment interactions are critical for optimizing cultivation practices for *M. savatieri*. Preliminary research indicates notable differences in rhizosphere microbiota between wild and cultivated stands, partially driven by soil nutrients (Li F. Q. et al., 2025; Luo et al., 2024). Nonetheless, current knowledge remains fragmented, and a systematic understanding of how varying forest stands affect the assembly, function, and ecological interactions of endophytic bacterial communities in *M. savatieri* is urgently needed.

To address these gaps in the literature, we investigated wild *M. savatieri* populations in four typical forest stands. By integrating 16S rRNA high-throughput sequencing, and multivariate statistics with comprehensive soil physicochemical profiling, we aimed to address the following questions: (1) How do plant organs and site-specific environmental conditions covary to shape the community structure and diversity of endophytic bacteria in *M. savatieri*? (2) In what ways do the community structure and functional potential of these endophytic communities vary among the four contrasting understory habitats? (3) What edaphic factors are most strongly associated with spatial divergence within these bacterial communities? By elucidating the multidimensional regulatory framework comprising organ-specific microenvironments, microbial interactions, stand filtering, and soil properties that govern the assembly and functional adaptation of the *M. savatieri* endophytic bacteriome, we aim to provide a scientific basis for the conservation and targeted microecological management of this rare medicinal species during understory cultivation.

## 2 Materials and methods

### 2.1 Study site description

Site selection was based on previous surveys of natural population distribution of *M. savatieri* to ensure ecological representativeness. In this study, typical understory wild habitats within the natural distribution range of *M. savatieri* were selected as sampling sites. Four representative forest stands were established:

Site 1 was located in Dongyuan Village, Fenyi County, Jiangxi Province (114.76°E, 27.86°N). The area has a mid-subtropical humid monsoon climate, with a mean annual temperature of 17.9 °C, an extreme maximum of 40.0 °C, and an extreme minimum of −7.2 °C. The frost-free period lasts approximately 270 days, with an annual sunshine duration of 1,624 h and a mean annual precipitation of 1,666.8 mm. The stand type is a *Gardenia jasminoides* plantation, and the soil is predominantly mountain yellow-brown soil. The site is under intensive management, with pruning and fertilization applied 2–3 times per year.

Site 2 was located at Wugongshan Forest Farm, Anfu County, Jiangxi Province (114.18°E, 27.82°N), in a subtropical hilly zone. The mean annual temperature is 18.3 °C, with an extreme maximum of 39.7 °C and an extreme minimum of −8.3 °C. The frost-free period lasts approximately 279 days, with an annual sunshine duration of 1,649 h and mean annual precipitation of 1,562.1 mm. The stand type is a *Pinus massoniana* plantation, and the soil is low-mountain hilly yellow-brown soil. The site is under light management, with fertilization and tending each applied once per year.

Site 3 was located in Xiaojiang Town, Xinfeng County, Jiangxi Province (114.92°E, 25.39°N). The area has a south-subtropical monsoon climate, with a mean annual temperature of 19.7 °C, an extreme maximum of 40.0 °C, and an extreme minimum of −4.0 °C. The frost-free period lasts approximately 280 days, with an annual sunshine duration of 1,810.7 h and mean annual precipitation of 1,502.3 mm. The stand type is a *Camellia oleifera* forest, and the soils are primarily red soil and red-yellow soil developed from granite and shale. The site is under extensive management, with no management activities for the past five consecutive years.

Site 4 was located in a *Phyllostachys edulis* forest in Jingning County, Zhejiang Province (119.13°E, 28.08°N). The area has a mid-subtropical monsoon climate, with a mean annual temperature of 17.5 °C, an extreme maximum of 40.5 °C, and an extreme minimum of −8.3 °C. The frost-free period lasts approximately 241 days, with an annual sunshine duration of 1,728.5 h and mean annual precipitation of 1,661.2 mm. The stand type is a *Phyllostachys edulis* forest, and the soils are predominantly red soil and red-yellow soil. The site is also under extensive management, with no management interventions for the past 5 years.

The geographical, climatic, and stand characteristics for each site are presented in Table 1. Each stand type was represented by a single geographic site, with three plots per site serving as within-site replicates. Therefore, in our analyses, we treat the “site” as the unit of habitat classification, and the results are interpreted as associations with the integrated site-specific environment. Host plants were identified by field observation (Zhang et al., 2015; Shi et al., 2022), but the host identity was not systematically quantified and was excluded from analysis.

**TABLE 1:** Basic situation of *M. savatieri* sampling locations.

Sample	Site	Mean annual temperature (°C)	Mean annual precipitation (mm)	Stand type	Soil type
Pi	Anfu, Jiangxi	18.3	1562.1	*Pinus massoniana*	Yellow-brown soil
Ga	Fenyi, Jiangxi	17.9	1666.8	*Gardenia. jasminoides*	Yellow-brown soil
Ca	Xinfen, Jiangxi	19.7	1502.3	*Camellia oleifera*	Red soil, Red-yellow soil
Ph	Jingning, Zhejiang	17.5	1661.2	*Phyllostachys edulis*	Red soil, Red-yellow soil

### 2.2 Sample collection and pre-processing

Across all forest stand types, sampling was systematically conducted during the active growth phase of *M. savatieri* (March–April 2025). Three independent biological replicate plots were established per site, separated by at least 50 m to ensure spatial independence. Within each plot, five healthy plants of comparable developmental stage (based on the stem height and leaf number) were pooled to generate one composite sample, and the same five plants were used for both aboveground (stems and leaves, pooled in equal mass) and belowground (roots) tissue collection. Rhizosphere soil samples (depth of 0–20 cm) were collected by carefully uprooting the plants and gently shaking off adhered adhering soil, after which the soil tightly adhered to the roots was collected using a sterile brush. For each plot, soil from the five plants was pooled into one composite sample.

The harvested plants were separated into aboveground (stems and leaves) and belowground (roots) tissues. The root tissues were collected as whole root systems without distinguishing between haustorial and non-haustorial portions, as such dissection would compromise the tissue integrity and hinder DNA extraction owing to the small size and delicate nature of *M. savatieri* roots. To remove the adhered soil, tissues were gently agitated and brushed with sterile brushes and subsequently rinsed in sterile deionized water for 30 s. Surface sterilization was conducted by sequential immersion in 70% ethanol for 2 min, 2.5% NaClO (with 0.1% Tween-80) for 5 min, and subsequently in 70% ethanol for 30 s, followed by three rinses with sterile deionized water. The sterilization efficacy was verified by plating 100 μL of the final rinse onto Luria–Bertani (LB) agar medium and incubating at 28 °C for 72 h. Samples showing microbial growth were re-sterilized and re-tested; after up to three failed attempts, a reserve replicate was substituted. Only after all samples passed validation were tissues processed further, ensuring three validated replicates per site-tissue combination. Sterilized tissues were aliquoted, flash-frozen in liquid nitrogen, and stored at −80 °C until DNA extraction.

Polymerase chain reaction (PCR)-negative controls (molecular-grade water replacing the template DNA) were included in each amplification run to monitor reagent contamination during PCR. Sterile water from the final rinsing step was plated onto LB agar to verify the surface sterilization efficacy, as described above. Sequences assigned to chloroplasts, mitochondria, archaea, and unclassified taxa at the domain level were removed bioinformatically prior to downstream analysis to minimize the impact of potential contaminants.

### 2.3 Analysis of soil physicochemical properties

Soil pH was measured using a pH meter (Orion Star A211, Thermo Fisher Scientific, USA). Soil organic matter (OM) content was quantified via the potassium dichromate oxidation method (Li Y. H. et al., 2025). Total nitrogen (TN) content was determined using the Kjeldahl method, while available nitrogen (AN) was evaluated through the alkali diffusion technique (Zuo et al., 2026). Total potassium (TK) was measured by sodium hydroxide fusion followed by flame photometry, and available potassium (AK) was extracted using ammonium acetate and flame photometry (Lan et al., 2024). For total phosphorus (TP), samples were subjected to sodium hydroxide fusion, and concentrations were determined using the molybdenum antimony spectrophotometric method; available phosphorus (AP) was extracted with an ammonium fluoride–hydrochloric acid solution and analyzed by the molybdenum antimony colorimetric method (Zhou et al., 2024). All analyses were performed in triplicate.

### 2.4 Microbial DNA extraction, amplification, and sequencing

All microbial DNA extraction and sequencing services were performed by Convinced-Test Co., Ltd., (Nanjing, China). Total genomic DNA was extracted from plant tissues using the cetyltrimethylammonium bromide (CTAB) method. The bacterial community analysis targeted the V3–V4 hypervariable regions of the 16S rRNA gene. PCR amplification was performed on an Applied Biosystems GeneAmp^®^ 9700 thermal cycler (Thermo Fisher Scientific) using barcoded specific primers 341F (5′-CCTACGGGNGGCWGCAG-3′) and 806R (5′-GGACTACNNGGGTATCTAAT-3′). Each PCR run included negative controls (molecular-grade water replacing template DNA) to monitor reagent contamination. Each 20-μL PCR reaction comprised 4 μL of 5 × FastPfu Buffer, 2 μL of 2.5 mM dNTPs, 0.8 μL each of 5 μM forward and reverse primers, 0.4 μL of FastPfu Polymerase, approximately 10 ng of template DNA, and ddH2O to reach the final volume. Thermal cycling included an initial denaturation at 95 °C for 5 min, followed by 27 cycles of 95 °C for 30 s, 58 °C for 30 s, and 72 °C for 45 s, with a final extension at 72 °C for 10 min. PCR products were visualized on a 2% agarose gel, and correctly sized amplicons were purified with the AxyPrep DNA Gel Recovery Kit (Thermo Fisher Scientific). Equal amounts of purified amplicons from all samples were pooled and subjected to paired-end (PE250) sequencing on the Illumina NovaSeq platform.

### 2.5 Bioinformatics analysis

Raw sequencing reads were quality-filtered using Trimmomatic (version 0.33) with a 10-bp sliding window and a Q20 quality threshold; reads shorter than 50 bp after trimming were discarded. Paired-end reads were merged using FLASH (version 1.2.7) with a minimum overlap of 10 bp and a maximum mismatch ratio of 0.2 in the overlapping region; unmerged reads were discarded. Sequences were assigned to individual samples based on their specific barcodes and primers, allowing zero mismatches for barcodes and up to two mismatches for primers; sequence orientation was corrected accordingly. Chimeric sequences were detected and removed using the UCHIME algorithm implemented in USEARCH (version 10.0), employing both *de novo* and reference-based approaches against the Genomes OnLine Database (GOLD) database. Quality filtering and chimera removal resulted in an average of 46,127 ± 5,834 (mean ± SD) high-quality reads per sample, with chimeric reads accounting for 3.8% ± 0.6% of the quality-filtered reads across all samples. Non-target sequences, including chloroplasts, mitochondria, archaea, and unclassified taxa at the domain level, were removed using the subset_taxa function in QIIME v1.9.1. Only bacterial sequences classified at the phylum level or below were retained for downstream analyses.

Operational taxonomic units (OTUs) were clustered at a 97% similarity threshold using UPARSE (version 7.1). OTU clustering at 97% similarity was chosen to maintain consistency with previous studies on *M. savatieri* and related hemiparasitic plants (Li F. Q. et al., 2025; Luo et al., 2024; Zhang et al., 2024), enabling direct comparability of the results across studies. Although amplicon sequence variant (ASV)-based methods (e.g., DADA2 or Deblur) offer improved taxonomic resolution and reproducibility, the OTU approach at 97% similarity remains widely accepted for the analyses of community-level diversity and composition. Taxonomy was assigned to each OTU representative sequence using the RDP Classifier (version 2.2) against the SILVA database (release 132) with a confidence threshold of 70%. Multiple sequence alignment of OTU representative sequences was performed using MAFFT (version 7.0), and a phylogenetic tree was constructed using FastTree (version 2.1.10) with the GTR model. Taxonomic composition at different classification levels was visualized using the “ggplot2” package in R (version 4.0.0).

### 2.6 OTU table summary

Operational taxonomic unit relative abundance per sample was calculated as the number of reads assigned to an OTU divided by total filtered reads. OTU representative sequences were selected as the most abundant sequence within each OTU cluster. For genus-level analysis, relative abundances were obtained by summing OTU relative abundances assigned to the same genus (RDP confidence ≥70%). To account for the uneven sequencing depth, all samples were rarefied to 24,000 reads per sample before calculating the alpha and beta diversity metrics. Rarefaction curves were generated for each sample using the rarecurve function in the vegan package of R to confirm that the sequencing depth was sufficient to capture most of the species diversity. Good’s coverage was calculated for each sample using Mothur and ranged from 97.9% to 99.2% across all samples, with a mean value of 98.6% ± 0.4%, confirming that the sequencing depth adequately represented the bacterial communities. A total of 12,497 OTUs were retained after quality filtering and used for all subsequent diversity and community composition analyses.

Alpha diversity indices, including observed OTU species (OTU count), Shannon-Wiener index (*H*), Pielou evenness index, and Faith’s phylogenetic diversity (*PD*) index, were calculated for each sample using Mothur (v1.44.3). Statistical comparisons of the alpha diversity between tissue types and among sites were conducted using two-way analysis of variance (ANOVA) with tissue type and site as the fixed factors, followed by Tukey’s honestly significant difference (HSD) *post-hoc* tests for pairwise comparisons. All *p*-values were adjusted for multiple testing using the Benjamini-Hochberg false discovery rate (FDR) method at *q* < 0.05. A Bray-Curtis distance matrix was computed from the OTU relative abundance data for beta diversity analysis. Non-metric multidimensional scaling (NMDS) was conducted for ordination. Permutational multivariate ANOVA (PERMANOVA) was used to test for significant differences in the community structure and to partition the variation among the compartment, site, and their interaction.

Data processing and visualization were conducted using the Tutools platform^1^ and Wekemo Bioincloud^2^. The figures were refined using Adobe Illustrator software.

### 2.7 Functional prediction

Functional predictions were performed using PICRUSt2 (version 2.5.2) with the default parameters. Predicted gene families were collapsed to KEGG Orthology (KO) numbers and further summarized at KEGG Pathways Level 1–3. Only pathways with a total relative abundance >0.01% across all samples were retained for downstream analysis. The predicted Kyoto Encyclopedia of Genes and Genomes (KEGG) pathway abundances were normalized using the built-in PICRUSt2 normalization algorithm. Statistical comparisons of the pathway abundances between compartments and among sites were conducted using Kruskal–Wallis tests followed by pairwise Wilcoxon rank-sum tests. All *p*-values were corrected for multiple testing using the Benjamini–Hochberg (FDR) method at *q* < 0.05.

### 2.8 Differential abundance analysis

Differential abundance analysis was conducted using linear discriminant analysis effect size (LEfSe) to identify the significantly enriched genera among tissue types (aboveground vs. belowground) and forest stands (four sites). We used the Kruskal–Wallis test (α = 0.05) for initial group comparisons, followed by pairwise Wilcoxon tests to identify the specific group differences. A logarithmic linear discriminant analysis (LDA) score threshold of 4.0 was applied to retain the biologically meaningful biomarkers. Subclass specification was not applied, as the design focused on the main effects of the tissue and site rather than on paired or nested structures. Taxa with a mean relative abundance < 0.01% across all samples were excluded to reduce the noise. The Benjamini–Hochberg (FDR) correction at *q* < 0.05 was applied to pairwise comparisons to control for multiple testing.

### 2.9 Statistical analysis

Soil physicochemical data are presented as mean ± standard deviation (SD). Differences in rhizosphere soil physicochemical properties among forest stands were evaluated using Duncan’s multiple range test in SPSS 25.0 (SPSS, Inc., Chicago, IL, USA), with different lowercase letters indicating statistically significant differences (*p* < 0.05).

Mantel tests were conducted to evaluate the overall correlations between the soil physicochemical property matrix and the microbial community structure matrix (genus-level relative abundance), as well as between the soil property matrix and the predicted functional profile matrix (KEGG Level 3). Correlation heatmaps based on Spearman’s correlation coefficients were generated to visualize the strength and direction of relationships between individual soil factors and key bacterial genera or functional profiles.

Redundancy analysis (RDA) was used to quantify the variation in bacterial community structure and functional profiles explained by the soil physicochemical properties, using Hellinger-transformed genus-level relative abundance and KEGG level 3 profiles as the response variables and soil factors as the explanatory variables. Forward selection with Monte Carlo permutation tests (9,999 permutations) identified variables significantly associated with the community structure (*p* < 0.05). Variables with a variance inflation factor (VIF) of >10 were excluded; OM was removed owing to collinearity with TN (VIF = 12.3), and all retained variables had a VIF of <5. Each RDA model was based on 12 independent observations per tissue type; soil samples were paired with both above- and belowground tissues from the same plot to assess the correlations across compartments. Both marginal and conditional tests were significant for all retained variables (*p* < 0.05). The proportion of constrained (explained) and unconstrained variation is reported for each model. RDA was conducted using Canoco v5.0.

To formally evaluate the relative contributions of the plant compartment, site, and their interaction to the overall variation in the endophytic bacterial community structure, we conducted (PERMANOVA) based on Bray–Curtis dissimilarities using the adonis2 function in the package vegan of R (v2.6-4) (Oksanen et al., 2022). The model was specified as distance ∼ compartment site, with permutations restricted within plots (strata = Plot) to account for the nested design (three plots per site). The significance of each term (compartment, site, and their interaction) was tested with 9,999 permutations. To determine whether significant PERMANOVA results can be attributable to the differences in multivariate dispersion rather than to genuine shifts in the community composition, we conducted tests for homogeneity of multivariate dispersions (PERMDISP) using the betadisper function, followed by permutation-based ANOVA (9,999 permutations) on the distances to group centroids (Anderson, 2006).

## 3 Results

### 3.1 Soil physicochemical properties in different habitats

Significant differences were observed in the physicochemical properties of rhizosphere soils among the four forest stands (*p* < 0.05; Table 2). All soils were acidic, with varying degrees of acidity. The *C. oleifera* and *P. eduli*s stands demonstrated higher pH values (5.29 and 5.10, respectively), whereas soils in the *P. massoniana* and *G. jasminoides* stands were the most acidic (pH 4.40 and 4.32, respectively). Both TK and AK concentrations were highest in the *P. edulis* stand, significantly exceeding those in the other stands. Additionally, soil OM content was highest in the P. edulis stand (47.85 g⋅kg^–1^), significantly greater than that in the *C. oleifera* stand. Elevated OM, TN, and AN contents were also observed in the *G. jasminoides* and *P. edulis* stands. The highest AP content was recorded in the *C. oleifera* and *P. edulis* stands. Collectively, these results suggest that the *P. edulis* stand possesses the highest overall soil fertility, whereas the *C. oleifera* stand exhibits relatively lower OM and TK contents.

**TABLE 2:** Physicochemical properties of the rhizosphere soil of *Monochasma savatieri* under different forest stands.

Index		Pi	Ga	Ca	Ph
pH		4.40 ± 0.07b	4.32 ± 0.22b	5.29 ± 0.16a	5.10 ± 0.03a
Total nutrient content (g⋅kg^–1^)	OM	41.40 ± 5.84a	47.37 ± 6.70a	28.27 ± 0.65b	47.85 ± 1.59a
	TN	1.66 ± 0.09b	2.07 ± 0.23a	1.00 ± 0.06c	1.86 ± 0.06ab
	TP	0.27 ± 0.01b	0.32 ± 0.09ab	0.40 ± 0.04a	0.22 ± 0.03b
	TK	12.32 ± 0.13d	13.98 ± 1.11c	20.33 ± 0.74b	25.49 ± 0.88a
Available nutrient content (mg⋅kg^–1^)	AN	102.37 ± 12.27b	144.90 ± 25.39a	128.25 ± 3.48ab	145.73 ± 2.94a
	AP	0.45 ± 0.03c	1.66 ± 0.73b	3.25 ± 0.35a	3.17 ± 0.69a
	AK	70.53 ± 5.03b	61.23 ± 6.95b	62.66 ± 3.37b	218.48 ± 8.69a

Data are presented as mean ± standard deviation (*n* = 3). Different lowercase letters within a column indicate significant differences among the stand types (*p* < 0.05). OM, organic matter; TN, total nitrogen; TP, total phosphorus; TK, total potassium; AN, available nitrogen; AP, available phosphorus; AK, available potassium.

### 3.2 Sequencing data and OTU overview

Based on high-throughput sequencing technology, bacterial communities were analyzed for all samples, comprising aboveground and belowground tissues of *M. savatieri* collected from four forest stands *(P. massoniana*, *G. jasminoides*, *C. oleifera*, and *P. edulis*). OTU clustering was performed at a 97% similarity threshold, yielding a total of 14,556 OTUs across all samples. Among these, 100% of the OTUs were successfully assigned to the phylum level, 85.85% (12,497 OTUs) to the genus level, while only 7.20% (1,048 OTUs) were assigned to the species level, reflecting the inherent limitation of the 16S rRNA gene V3–V4 region in resolving taxonomic relationships at the species level. The sequences obtained across all samples were classified into 28 phyla, 57 classes, 142 orders, 249 families, 696 genera, and 351 species. After quality filtering and chimera removal, an average of 46,127 ± 5,834 high-quality reads per sample were retained. The mean Good’s coverage across all samples was 98.6% ± 0.4% (range: 97.9%–99.2%), indicating that the sequencing depth was sufficient to capture most of the bacterial diversity in each sample.

### 3.3 Analysis of endophytic bacterial diversity in *M. savatieri* across habitats

Alpha diversity metrics–including observed OTU richness, Shannon index, Pielou evenness index, and Faith’s phylogenetic diversity (PD) index–revealed that both plant tissue type and sampling site significantly influenced the alpha diversity of endophytic bacterial communities associated with *M. savatieri* (*p* < 0.05; Figures 1a–d). In this study, samples were coded by species–*Pinus massoniana* (Pi), *Camellia oleifera* (Ca), *Phyllostachys edulis* (Ph), and *Gardenia jasminoides* (Ga)–and tissue type (A: aboveground; S: belowground).

**FIGURE 1:**
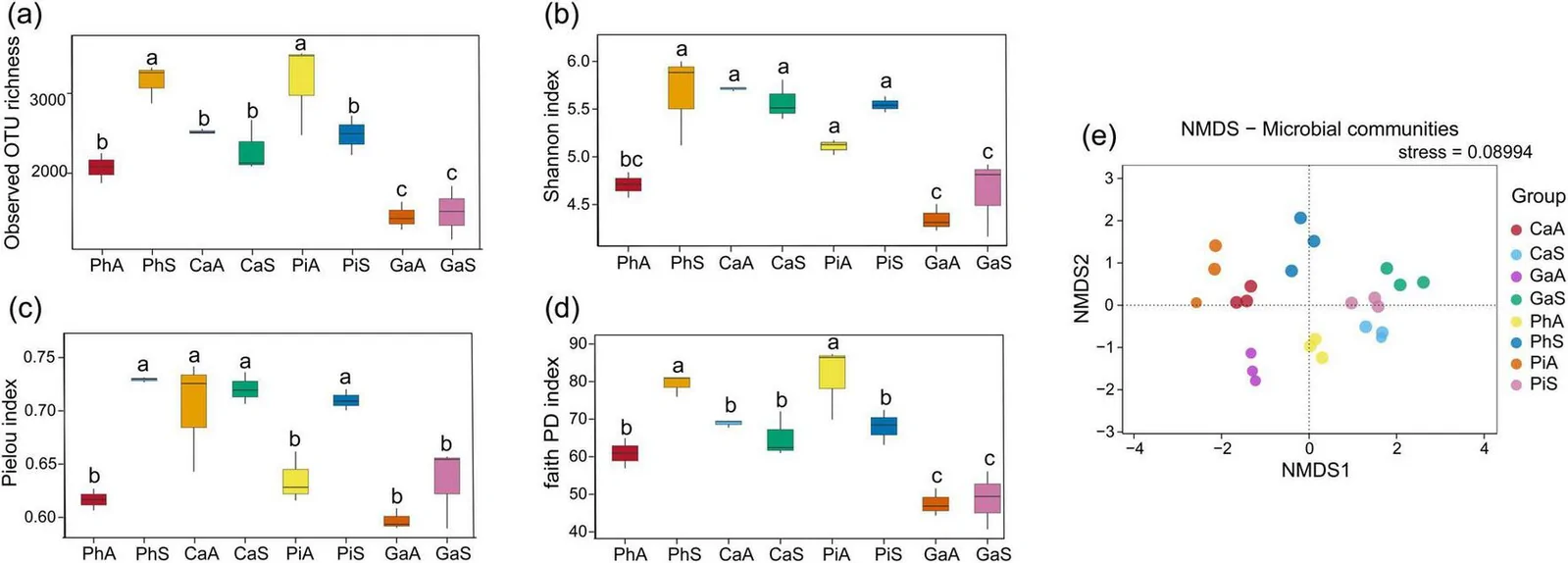
Comparative analysis of endophytic bacterial community diversity in *Monochasma savatieri* across different habitats. **(a)** Observed OTU richness index; **(b)** Shannon index; **(c)** Pielou evenness index; **(d)** Faith’s phylogenetic diversity (PD) index; **(e)** Non-metric multidimensional scaling (NMDS) ordination based on Bray–Curtis distances (stress = 0.089). Different letters above bars in panels **(a–d)** indicate significant differences among groups (*p* < 0.05). Stand type and tissue codes: Pi, *Pinus massoniana*; Ga, *Gardenia jasminoides*; Ca, *Camellia oleifera*; Ph, *Phyllostachys edulis*; A, aboveground compartment; S, belowground compartment.

Specifically, the diversity differences between aboveground and belowground tissues varied inconsistently among the four understory habitats: PhS exhibited significantly higher bacterial diversity (richness and Shannon index) than PhA (*p* < 0.05); PiA showed the highest observed OTU richness among all samples, significantly exceeding that of PiS and most other samples (*p* < 0.05), although its Shannon index and evenness were comparable to those of PiS (*p* > 0.05); no significant differences were observed between CaA and CaS across any diversity metric (*p* > 0.05). Under the same tissue condition, alpha diversity also varied significantly among forest stands: among belowground samples, PhS and PiS exhibited the highest diversity, with CaS intermediate and GaS the lowest; among aboveground samples, PiA showed the highest diversity, with PhA and CaA intermediate and GaA the lowest. PiA and PhS displayed the broadest phylogenetic diversity (Faith’s PD index), significantly exceeding that of other groups (*p* < 0.05). In contrast, the *G. jasminoides* stand (GaA and GaS) consistently exhibited the lowest values across all alpha diversity indices.

Non-metric multidimensional scaling based on Bray–Curtis distances clearly depicted the β-diversity of endophytic bacterial community structures across samples (Figure 1e). The model exhibited a good fit to the distance data, with a stress value of 0.089, which falls within the range considered acceptable for ecological ordination (stress < 0.1). Samples from different forest stands were distinctly separated in the ordination space, and the above- and belowground samples within each stand also showed a certain degree of separation, highlighting significant differences in the bacterial community structure among forest stand types. PERMANOVA revealed that compartment (*R*^2^ = 31.25%, *p* < 0.001), site (*R*^2^ = 24.70%, *p* < 0.001), and their interaction (*R*^2^ = 31.04%, *p* < 0.001) significantly influenced the endophytic bacterial community structure (Table 3). The compartment factor explained the largest proportion of variation, followed closely by the compartment × site interaction, whereas site alone explained the smallest proportion. These results indicate that although the plant-tissue type is a primary structuring force, its effects are strongly modulated by site-specific environmental conditions. The PERMDISP tests confirmed that the observed differences were not driven by unequal group dispersions (compartment: *F* = 0.0011, *p* = 0.9726; site: *F* = 0.04, *p* = 0.9893), indicating that the PERMANOVA results reflect genuine differences in the community composition rather than unequal group dispersions.

**TABLE 3:** Results of permutational multivariate analysis of variance (PERMANOVA) based on the Bray–Curtis distances for the endophytic bacterial communities of *Monochasma savatieri.*

Source	*Df*	Sum of sqs	*R* ^2^	*F*	*Pr* (>*F*)
Compartment	1	2.3402	0.3125	38.421	<0.001
Site	3	1.8494	0.2470	10.121	<0.001
Compartment × site	3	2.3240	0.3104	12.719	<0.001
Residual	16	0.9745	0.1302		
Total	23	7.4881	1.000		

Thus, although the belowground tissues tended to show higher diversity in certain habitats (e.g., *P. edulis*), this pattern was not universal: the aboveground tissues of *P. massoniana* exhibited the highest observed OTU richness among all samples. These results indicate that the direction and magnitude of tissue-specific diversity differences are habitat-dependent, precluding a general statement that belowground tissues uniformly harbor a higher diversity than aboveground tissues across all sites.

### 3.4 Composition of endophytic bacterial communities in *M. savatieri* across habitats

The flower plot revealed shared and unique OTUs among the four stands (Figure 2a). A total of 230 OTUs were common to all stands. PiA harbored the greatest number of unique OTUs (2,061), followed by PhS (1,646), whereas GaA contained the fewest unique OTUs (199). Venn diagrams provided further insights into the OTU overlap between aboveground and belowground tissues across stands (Figures 2b,c). Aboveground tissues shared 777 OTUs. Among these, *P. massoniana* displayed the greatest number of unique OTUs (2,692) and *G. jasminoides* the least (384). Notably, *P. massoniana* and *C. oleifera* shared numerous OTUs (1,310). For belowground tissues, 926 OTUs were shared among the stands. *P. edulis* contained the most unique OTUs (3,777), and G. *jasminoides* had the fewest OTUs (562).

**FIGURE 2:**
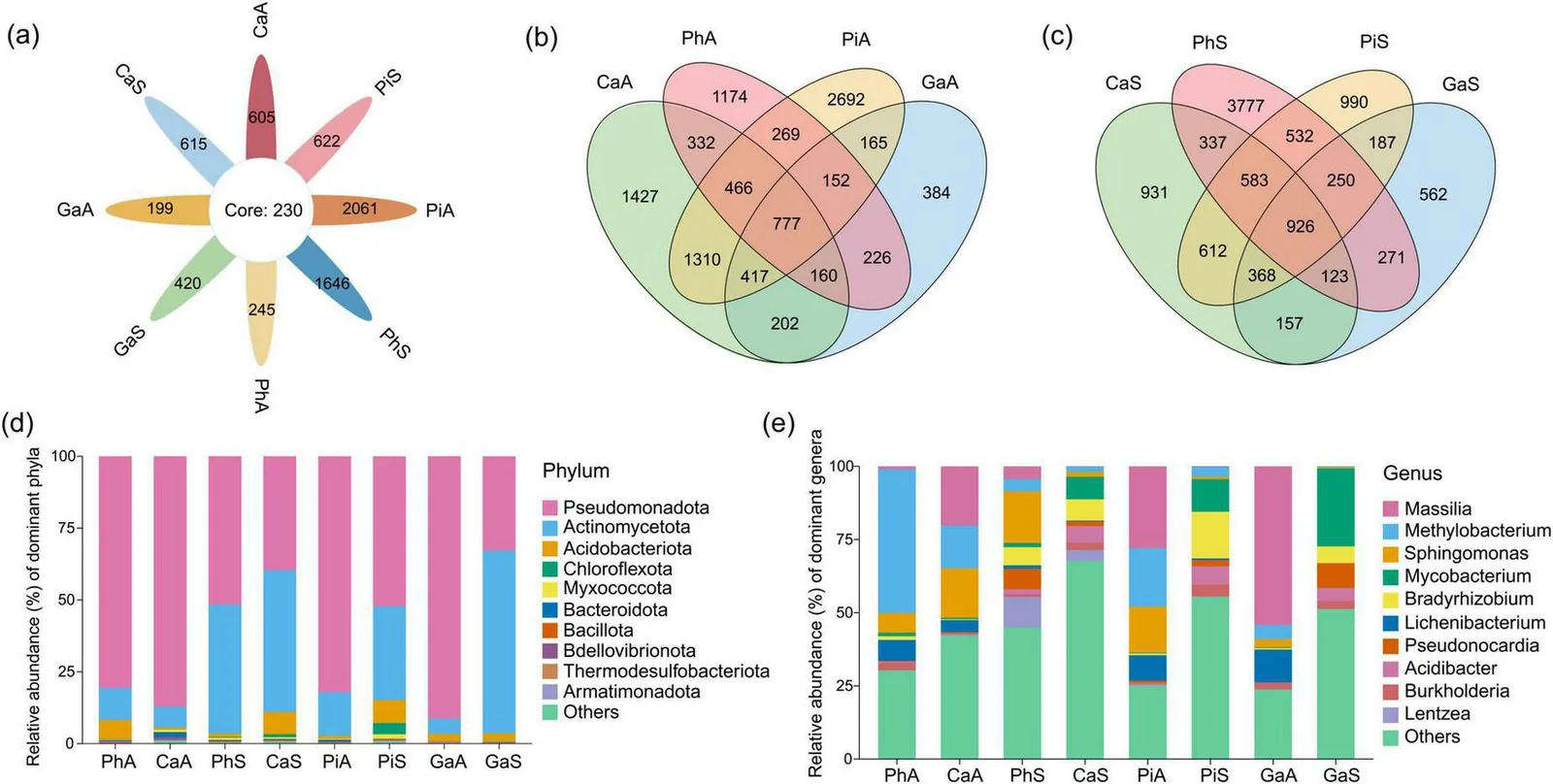
Composition of endophytic bacterial communities in *Monochasma savatieri* across different habitats. **(a)** Petal diagram showing shared and unique operational taxonomic units (OTUs) among the four stand types. **(b)** Venn diagram of OTUs in the aboveground parts. **(c)** Venn diagram of OTUs in the belowground parts. **(d)** Relative abundance of bacterial communities at the phylum level. **(e)** Relative abundance of the top 10 bacterial genera at the genus level.

The composition and relative abundance of endophytic communities were assessed at the phylum and genus levels (Figures 2d,e). At the phylum level, 28 bacterial phyla were identified, with Pseudomonadota, Actinomycetota (formerly Actinobacteria), and Acidobacteriota being the most prevalent. Notable differences in relative abundance were observed across samples; specifically, Pseudomonadota exhibited its highest relative abundance in GaA (91.29%) and lowest in GaS (32.76%). Conversely, Actinobacteriota were the most abundant in GaS (63.71%) and the least abundant in GaA (5.35%), while Acidobacteriota peaked in PiS (7.97%) and were lowest in CaA (0.73%). Overall, Pseudomonadota was more enriched in aboveground tissues, whereas Actinomycetota was enriched in belowground tissues.

Across all stands, 696 bacterial genera were detected. The 10 most abundant genera were selected for further analysis (Figure 2e). The dominant genera in the aboveground tissues were relatively consistent across stands and primarily included *Sphingomonas*, *Methylobacterium*, *Massilia*, and *Lichenibacterium*. Among these, *Massilia* reached its highest relative abundance in the *G. jasminoides* stand (54.19%), while *Pseudonocardia* was notably more prevalent in the *P. edulis* stand (49.16%) compared with other stands. In contrast, the composition of dominant genera in belowground tissues varied substantially. Dominant genera included *Mycobacterium*, *Pseudonocardia*, *Bradyrhizobium*, and *Acidibacter*; *Mycobacterium* reached its highest abundance in GaS (26.61%), and *Bradyrhizobium* was most abundant in PiS (15.94%), each significantly exceeding its representation in other stands.

### 3.5 Identification of key microbial taxa

Linear discriminant analysis effect size analysis (LDA > 4.0; FDR-adjusted *q* < 0.05) was conducted to identify differentially abundant microbial taxa within the endophytic communities of *M. savatieri* across various stands. The phylogenetic relationships among these taxa are depicted in the corresponding cladogram (Figure 3a), while the LDA score plot (Figure 3b) provides a quantitative assessment of their enrichment. The findings demonstrated distinct enrichment patterns specific to both stand and tissue types: PiS was enriched in Acidobacteriota (relative abundance: 7.97%), Chloroflexota (3.91%), and *Bradyrhizobium* (15.94%); PhS was characterized by Actinomycetota (44.94%) and genera such as *Sphingomonas* (17.65%), *Pseudonocardia* (6.95%), *Lentzea* (10.39%), and *Kutzneria* (3.46%); GaS was enriched in *Mycobacterium* (26.61%) and *Amycolatopsis* (9.98%), whereas GaA was enriched in *Massilia* (54.19%) and *Lichenibacterium* (11.21%); CaA featured *Pseudomonas* (3.50%) and *Rhizobacter* (4.32%) as biomarker taxa; and CaS was significantly enriched in *Actinocorallia* (12.78%) and *Acidothermus* (5.18%).

**FIGURE 3:**
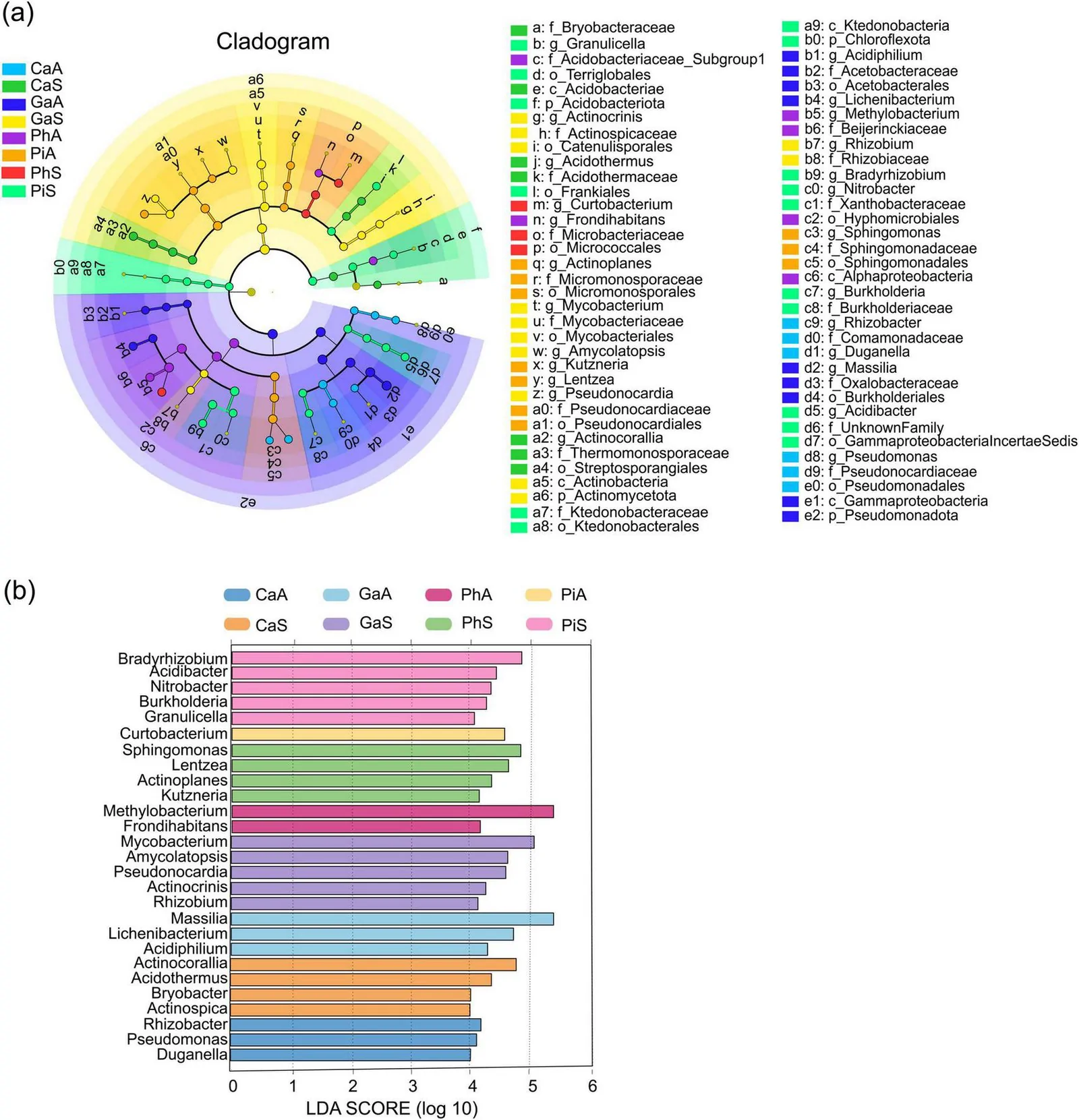
Identification of differentially abundant microbial taxa using linear discriminant analysis effect size (LEfSe). **(a)** Cladogram showing the phylogenetic distribution of taxa in different habitats (LDA score > 4.0). **(b)** Histogram of LDA scores for significantly enriched genera.

### 3.6 Predicted functional potential of endophytic bacteria in *M. savatieri*

The functional potential of endophytic bacterial communities within *M. savatieri* was predicted using PICRUSt2, leveraging 16S rRNA gene sequence data (Figure 4). PICRUSt2-based functional predictions yielded a mean (NSTI) of 0.12 ± 0.05 (range: 0.06–0.24), indicating that reference genomes were closely related to the community members in our samples. Analyses revealed that the predicted functions were primarily enriched in key categories, including metabolism, genetic information processing, and environmental information processing. Among these, metabolic functions constituted the largest proportion across all samples and were generally more abundant in aboveground tissues–a trend most evident in the *G. jasminoides* stand.

**FIGURE 4:**
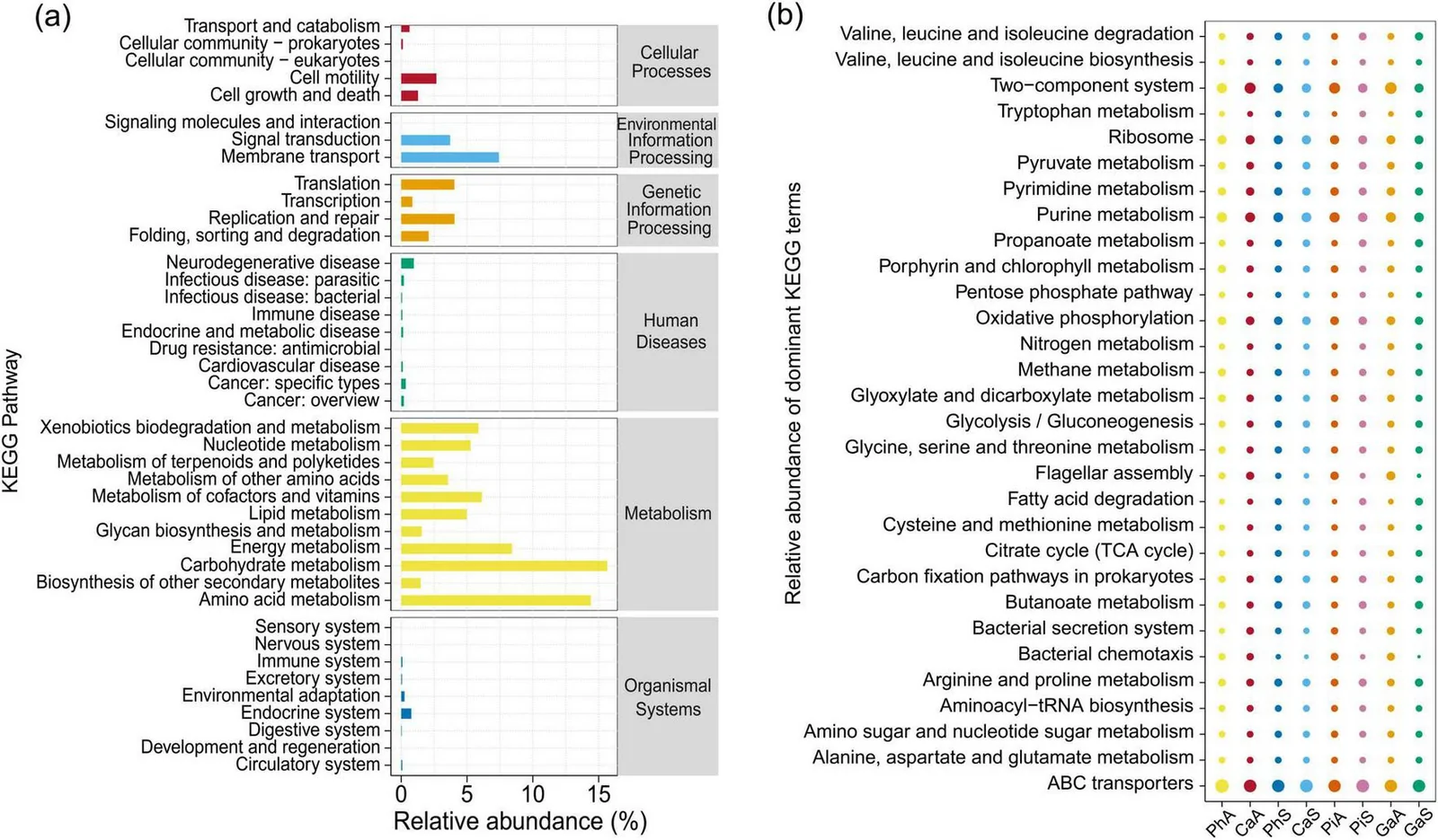
Functional prediction of endophytic bacterial communities based on PICRUSt2. **(a)** Relative abundance of predicted KEGG pathways at Level 1 and Level 2. **(b)** Bubble plot showing the relative abundance of selected KEGG pathways at Level 3 across different samples; bubble size reflects relative abundance.

A comparative analysis of predicted KEGG Level 3 pathway abundances demonstrated substantial variation in functional potential among forest stands and tissue types, indicative of adaptive strategies for distinct microenvironments. The aboveground tissues consistently showed higher inferred abundance of pathways associated with signal transduction (e.g., the two-component system), motility (e.g., flagellar assembly and bacterial chemotaxis), and secretion systems. Conversely, belowground tissues showed higher inferred abundance of pathways involved in amino acid degradation, fatty acid catabolism, and pyruvate metabolism, suggesting a greater representation of catabolic potential within root-associated communities.

The distinct patterns in inferred pathway representation among stands were also evident. For instance, PhA exhibited higher abundances of pathways related to porphyrin, chlorophyll, and methane metabolism, whereas GaS exhibited the highest abundance of tryptophan metabolism. CaS and PiS had comparable profiles across most degradation pathways, which was potentially attributable to the strongly acidic soils at these sites. Despite these specific variations, core pathways including ATP-binding cassette (ABC) transporters, purine metabolism, ribosomes, and oxidative phosphorylation, remained consistently abundant across all samples, reflecting the conservation of the predicted core functional potential across diverse environmental contexts.

### 3.7 Relationship between bacterial genera and soil properties

To quantitatively evaluate the associations among soil properties, microbial composition, and functional potential, soil physicochemical indices, key functional profiles (KEGG Level 3), and major bacterial genera identified by LEfSe analysis with LDA > 4.0 were selected. Mantel tests were conducted independently for aboveground and belowground tissues (Figures 5a,d). The findings demonstrated considerable variation in the strength and pattern of the associations between soil factors and biological variables across plant tissues. Specifically, the Mantel test showed that the major bacterial genera and functional profiles (KEGG Level 3) of the aboveground tissues were significantly correlated with TK and AK (*p* < 0.001).

**FIGURE 5:**
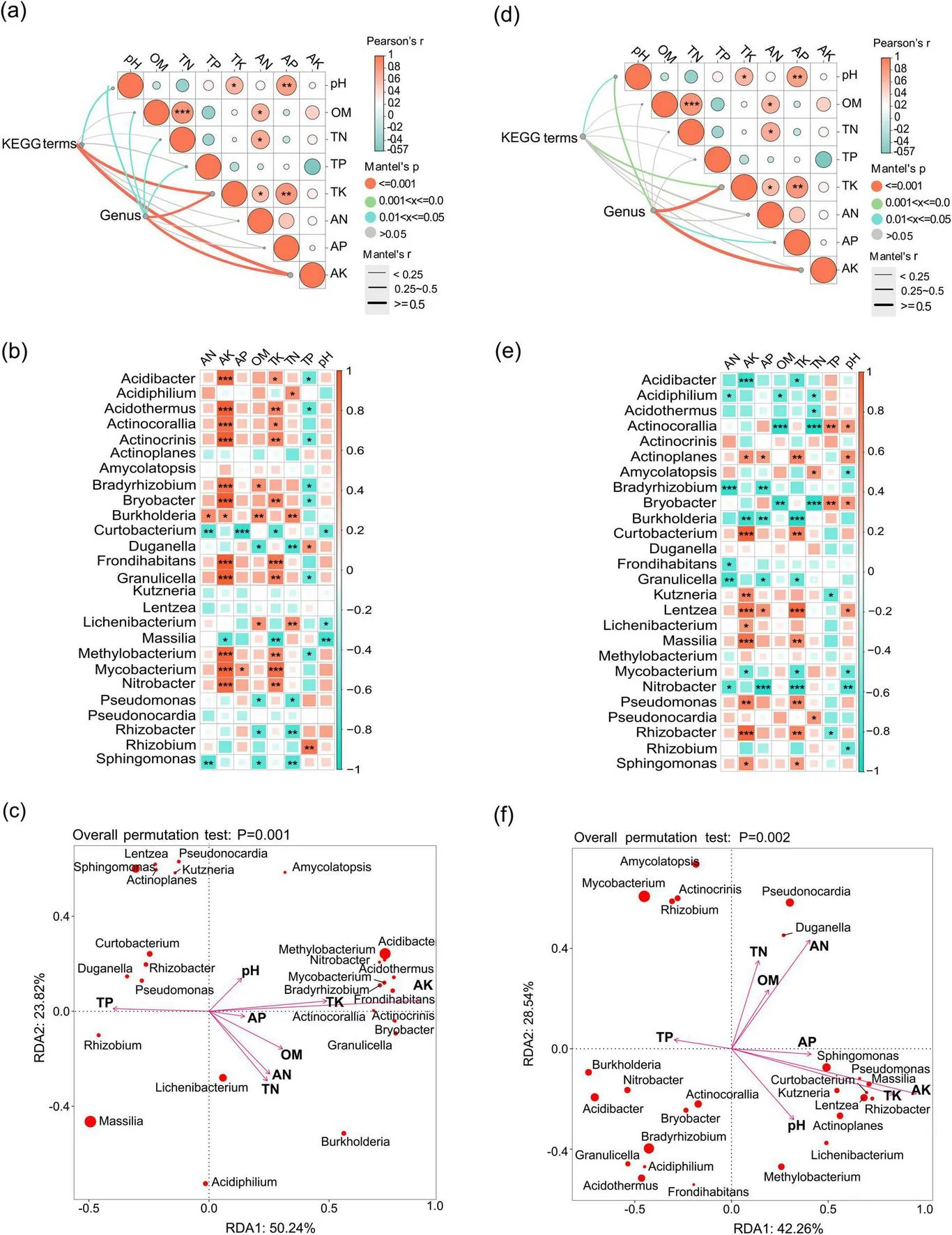
Correlation analysis between key bacterial genera and soil physicochemical properties in the endophytic communities of *Monochasma savatieri*. **(a)** Mantel test analyzing relationships among aboveground bacterial functional prediction profiles, key genera, and soil properties; line width indicates the Mantel’s “r” statistic, and asterisks denote statistical significance (*p* < 0.05). **(b)** Heatmap of Spearman’s rank correlation coefficients between key aboveground genera and soil properties. **(c)** Redundancy analysis (RDA) ordination showing the influence of soil properties on the composition of aboveground key genera; percentages on axes indicate explained variance. (**d–f**) Corresponding analyses for the belowground compartment [**(d)** Mantel test; **(e)** Spearman correlation heatmap; **(f)** RDA]. Significance levels: *p < 0.05, **p < 0.01, ***p < 0.001.

Redundancy analysis revealed significant associations between the soil properties and endophytic community structure (aboveground: *p* = 0.001; belowground: *p* = 0.002) with tissue-specific patterns. In aboveground tissues, RDA1 accounted for 50.24% of the variation, with AK and TK as the primary influencing factors (Figure 5c). TP was strongly associated with the negative RDA1 direction. Regarding the genus distribution, *Frondihabitans*, *Bradyrhizobium*, *Acidibacter*, and *Mycobacterium* were positively correlated with AK and TK, whereas *Pseudonocardia*, *Sphingomonas*, and *Lentzea* were negatively correlated. In the belowground tissues, RDA1 explained 42.26% of the total variation, with TK and AK emerging as the most strongly associated factors (Figure 5f). Genera such as *Lentzea*, *Massilia*, *Pseudomonas*, and *Rhizobacter* were positively correlated with these factors, whereas *Acidibacter*, *Burkholderia*, and *Nitrobacter* were negatively correlated, consistent with the Mantel test results. Correlation heatmaps further substantiated the magnitude and direction of Spearman correlations between the key bacterial genera and soil factors (Figures 5b,e).

### 3.8 Relationship between predicted functions and soil properties

Mantel tests, correlation heat maps, and RDA were employed to evaluate the impact of soil properties on the functional profiles of endophytic communities (Figure 6). The findings demonstrated that soil factors significantly affected the distribution of functional genes (aboveground RDA: *p* = 0.011; belowground RDA: *p* = 0.047), with distinct functional modules and organ specificity evident in the driving effects.

**FIGURE 6:**
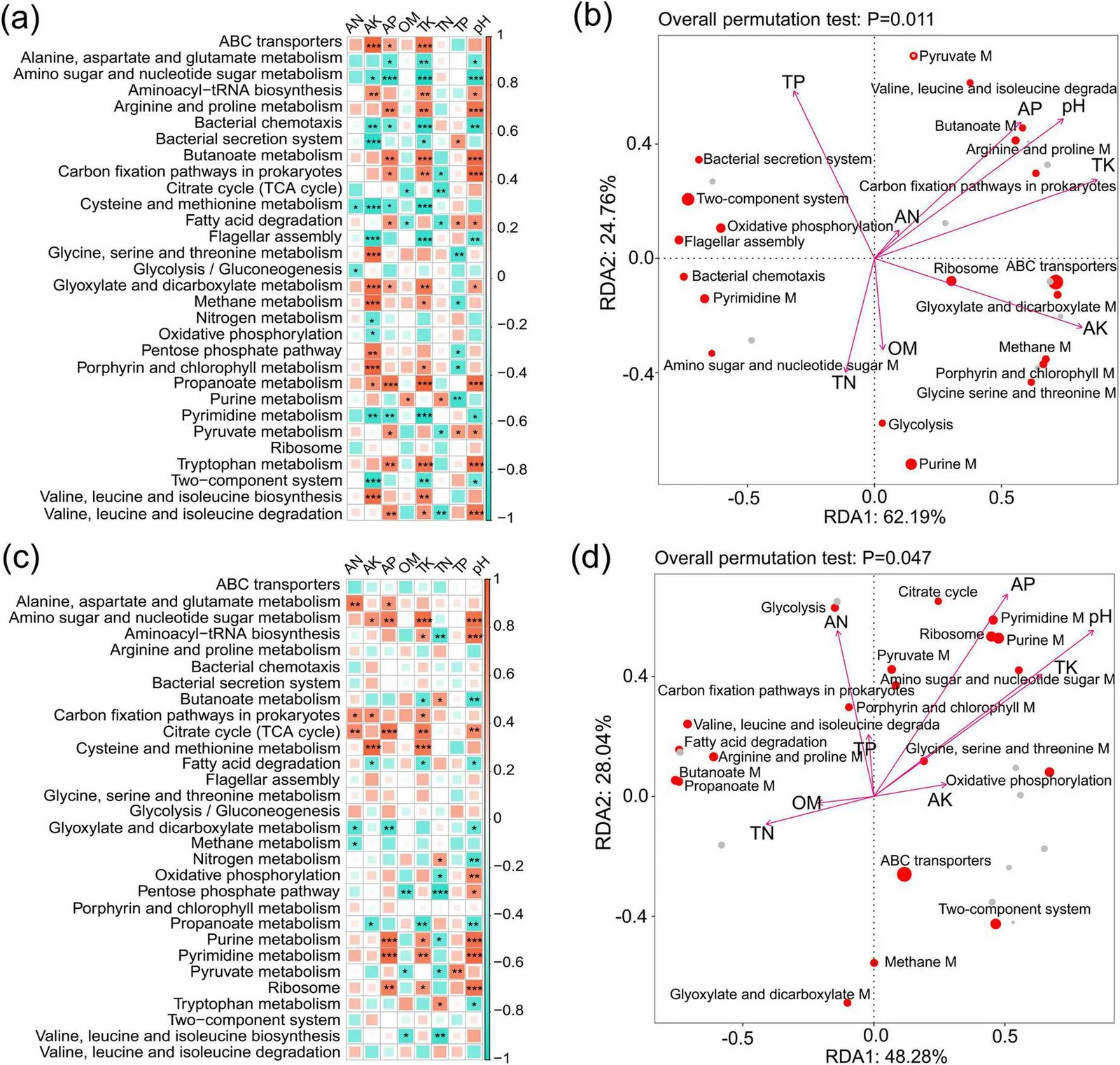
Correlation analysis between predicted functional profiles of endophytic bacteria and soil physicochemical properties. **(a)** Heatmap of Spearman correlations between aboveground functional pathways (KEGG Level 3) and soil properties. **(b)** Redundancy analysis (RDA) of aboveground functional profiles constrained by soil properties. **(c)** Heatmap of Spearman correlations between belowground functional pathways and soil properties. **(d)** RDA of belowground functional profiles constrained by soil properties. In RDAs, percentages on axes indicate the variance explained by the constrained ordination; M denotes metabolism. Significance levels: *p < 0.05, **p < 0.01, ***p < 0.001.

The RDA for aboveground tissues identified TK, AK, and pH as the main drivers (Figure 6b). Correlation analysis further revealed that AK was strongly positively correlated with pathways such as ABC transporters, methane metabolism, glyoxylate and dicarboxylate metabolism, and valine, leucine, and isoleucine biosynthesis. Meanwhile, TK and pH were positively correlated with degradation pathways, such as butanoate, propanoate, and tryptophan metabolism. In belowground tissues, the first two RDA axes accounted for 76.32% of the functional variation, with pH, TK, AP, and AN emerging as the primary contributors (Figure 6d). Additionally, pH, TK, and AP were positively correlated with the citrate cycle, purine metabolism, pyrimidine metabolism, and ribosome function. AN was closely related to energy metabolism processes, including alanine, aspartate, and glutamate metabolism and glycolysis.

Overall, the soil factors associated with the functional potential of endophytes demonstrated both distinct functional modules and organ-specificity. The potassium content (including TK and AK) was among the strongest correlates in both the aboveground and belowground environments.

## 4 Discussion

### 4.1 Organ microenvironment as the principal determinant of endophytic community structure and functional differentiation

Distinct differences were observed in the composition and diversity of endophytic bacterial communities associated with *M. savatieri* among the four habitats, and the differentiation pattern between the aboveground and belowground organs was consistent with the ecological niche theory of plant endophytes (Zhang et al., 2024). Formal variance partitioning through PERMANOVA confirmed that compartment explained the largest proportion of variation, followed by the compartment × site interaction, and site alone . The significant and substantial interaction term indicates that the effect of plant tissue on the community composition is strongly context-dependent, varying across the four understory habitats. However, the effect of tissue type on diversity varied among habitats: in the *P. edulis* stand, belowground tissues exhibited significantly greater OTU richness and Shannon diversity than their aboveground counterparts, whereas in the *P. massoniana* stand, aboveground tissues showed the highest observed OTU richness among all samples. The broadest phylogenetic diversity (Faith’s PD index) was simultaneously observed in the aboveground tissues of the *P. massoniana* stand and the belowground tissues of the *P. edulis* stand. These results indicate that stand type and tissue type collectively shape the diversity patterns of endophytic bacteria in *M. savatieri*, highlighting the important role of stand-specific microenvironments in regulating plant-associated microbial community assembly.

As a hemiparasitic species, *M. savatieri* roots not only form direct nutrient-acquisition connections to the host via haustoria but also actively engage with the rhizosphere soil microenvironment, thereby accessing a broader spectrum of microbial colonizers (Chen et al., 2020b; Li F. Q. et al., 2025). Conversely, aboveground organs are subject to intense ultraviolet radiation and variable humidity, rely primarily on photoassimilates for their carbon supply, and occupy relatively homogeneous habitats. This results in more selective microbial community structures (Jin et al., 2014; Li et al., 2018).

Niche filtering was evident among dominant bacterial phyla. Pseudomonadota emerged as the predominant phylum in aboveground tissues, reaching a relative abundance of 91.29% (Deepa et al., 2024). Members of this phylum are recognized for their ability to rapidly utilize simple carbon sources (Chobert et al., 2025), an adaptation well-suited to the dynamic aerial environment characterized by frequent material exchange. In contrast, Actinomycetota were enriched in belowground tissues, favoring the degradation of complex organic compounds such as lignin and cellulose found in root exudates, thus conferring a competitive advantage in the nutrient-limited rhizosphere (Adigoun et al., 2025; Thiem et al., 2023). This pattern of Pseudomonadota dominance aboveground and Actinomycetota enrichment belowground is consistent with findings in other plants, including tobacco (Yang et al., 2024).

Functional prediction analyses revealed a complementary pattern in the inferred pathway representation, which we tentatively framed as “aboveground sensing-belowground degradation,” which is a testable hypothesis rather than an established functional division. Aboveground microbiota showed higher inferred abundances of pathways associated with signal transduction, flagellar assembly, and chemotaxis, which if functionally active, may enable responses to fluctuating environmental cues (Duan et al., 2024). Conversely, belowground communities showed higher inferred abundances of catabolic pathways, such as amino acid and fatty acid degradation, which may hypothetically facilitate energy and carbon acquisition from root exudates and soil OM (Chen et al., 2021). However, metagenomic, metatranscriptomic, metabolomic, or cultivation-based approaches are required to confirm whether these predicted differences reflect actual biological activity *in situ*.

### 4.2 Ecological adaptation and potential utility of habitat-associated functional microbiota

Endophytic bacterial communities of *M. savatieri* exhibited distinct enrichment of taxa across the four habitats. These differences likely reflect the combined effects of site-specific environmental pressures (e.g., soil nutrients and climate) and host-associated selection. For example, in the nutrient-poor soil of the *P. massoniana* stand, we observed a significant enrichment of nitrogen-fixing *Bradyrhizobium* (relative abundance reaching 15.94%), a genus that includes species capable of nitrogen fixation (Pei et al., 2023; Zhao et al., 2018). Although this observation is consistent with the hypothesis that *Bradyrhizobium* may contribute to nitrogen acquisition in this habitat, we did not directly measure the nitrogen fixation activity, and the presence of *Bradyrhizobium* alone does not confirm this functional activity. Similarly, *Sphingomonas* and *Pseudomonas* were enriched in the *P. edulis* stand, and strains within these genera have been reported to possess plant growth-promoting traits (Santoyo et al., 2016); however, strain-level or functional validation is required to confirm such activities in our system.

The habitat-specific enrichment patterns observed here are consistent with recent findings on the host-dependent nature of plant-microbe associations. Ji et al. (2026) investigated endophytic bacterial communities in senescing leaves and needles of 11 tree species in Central European temperate forests and found that the host tree type (broadleaved vs. coniferous) acted as a strong selective filter, driving the tree type-specific enrichment of particular bacterial taxa. Similarly, Chen et al. (2026) demonstrated that deterministic host-associated selection consistently shaped endophytic communities of the medicinal plant *Rheum palmatum* across different seasons. These studies support the broader pattern that plant-mediated filtering can result in habitat-specific microbial enrichment, which in the case of *M. savatieri* appears to be further modulated by site-level environmental conditions. Considering the substantial variation in the community composition and functional potential across sites, we subsequently investigated the key environmental factors associated with these ecological differences.

### 4.3 Soil potassium as a key environmental correlate of the soil-plant-microbe continuum

Soil physicochemical properties serve as critical abiotic determinants that profoundly influence the composition of plant-associated endophytic bacterial communities (Huang et al., 2025; Zhao et al., 2026). Through comparative analyses across four forest stand types, we identified soil potassium, namely, both total and available potassium, as the strongest environmental correlates of variation in the community structure and functional gene profiles of endophytic bacteria of *M. savatieri*. However, because potassium availability covaried with the site, vegetation type, and other environmental factors, these associations should be interpreted as correlative rather than as causal. These results expand upon previous work on the rhizosphere microbiome within the same ecosystem. For instance, Li F. Q. et al. (2025) demonstrated that soil total nitrogen, available potassium, available nitrogen, and organic matter collectively drive the differentiation of the rhizosphere bacterial community in *M. savatieri*. This shift in focus from the rhizosphere to the endosphere establishes a logical continuum: key soil nutrients–particularly potassium–successively modulate the rhizosphere microbial reservoir and selectively filter endophytic communities colonizing plant tissues. This hierarchical filtering process creates a multilevel regulatory network that facilitates host adaptation in complex forest environments (Zhang et al., 2025).

Notably, a substantial soil potassium gradient was observed across the four forest stands (e.g., 25.49 g⋅kg^–1^ in the *P. edulis* stand vs. 12.32 g⋅kg^–1^ in the *P. massoniana* stand). Accompanying this gradient, both the community composition and functional profiles exhibited systematic differences between high- and low-potassium sites. For instance, endophytic communities in potassium-rich habitats showed relatively higher abundances of pathways related to energy metabolism and cofactor synthesis, whereas those from potassium-limited environments displayed higher abundances of nutrient-acquisition functions (e.g., nitrogen fixation and phosphorus solubilization) and stress-response pathways (Chen et al., 2024). Similar bacteria-driven regulation of soil functionality has been reported in other ecosystems (Liang et al., 2026). These correlative patterns suggest a testable hypothesis that potassium availability may influence the functional niche differentiation among endophytic bacteria, potentially through a “soil K^+^-plant physiology-microbiome function” regulatory framework. However, considering the confounding of potassium with other site-level factors, this proposed framework requires experimental validation through controlled potassium-manipulation or common-garden experiments before any causal interpretation can be justified. Nonetheless, the strong associations revealed in this study provide a clear direction and a working model for subsequent mechanistic studies.

### 4.4 Limitations and future perspectives

Some limitations of this study should be acknowledged. First, as each forest stand was represented by a single geographic site, the vegetation type is inherently confounded with the location, climate, and soil properties. The observed habitat-associated patterns should therefore be interpreted as correlative rather than as causal; future studies with replicated sites per stand type are required to disentangle these factors. Second, our functional predictions were based on PICRUSt2 and represent the inferred potential rather than the measured activity; metagenomic or metatranscriptomic approaches are required to confirm functional expression. Third, we did not include sequenced extraction blanks or final-rinse controls, which is a consideration particularly for low-biomass aboveground tissues. Fourth, as a hemiparasitic plant, *M. savatieri* forms haustorial connections with host roots, but the host identity was not systematically quantified; this represents an unmeasured factor that may influence the belowground communities and should be addressed in future studies. Despite these limitations, our study provides robust evidence for tissue-specific and habitat-associated patterns across multiple sites and offers testable hypotheses for future experimental studies.

## 5 Conclusion

In this study, by integrating organ microenvironments, habitat-specific conditions, and soil drivers, we clarify the multilevel regulatory framework governing the assembly and functional adaptation of endophytic bacterial communities in *M. savatieri*. Quantitative variance partitioning through PERMANOVA revealed that the plant compartment, understory habitat, and their interaction collectively shaped community the composition, indicating that both organ-specific microenvironments and habitat-level filtering jointly determine the endophytic community assembly. The findings suggest a pattern of functional complementarity in predicted pathway representation between aboveground and belowground tissues, with aboveground and belowground communities showing higher inferred abundances of sensing- and belowground-related pathways, respectively; however, this interpretation remains a testable hypothesis that requires experimental validation.

Furthermore, the distinct endophytic assemblages observed across the four understory habitats suggest that site-integrated environmental conditions (including the vegetation, soil, and climate) are associated with the enrichment of particular bacterial taxa. Among the measured soil properties, the potassium content emerged as the strongest correlate of endophytic community variation, although this association is correlative and requires experimental validation through controlled manipulation studies. These results provide testable hypotheses considering the ecological drivers of endophytic community assembly in *M. savatieri* and identify candidate bacterial taxa and environmental correlates, –particularly soil potassium, –that warrant further investigation through isolation, inoculation, and controlled cultivation experiments to determine their functional significance and potential applications.

## Data Availability

The datasets presented in this study can be found in online repositories. The names of the repository/repositories and accession number(s) can be found below: https://www.ncbi.nlm.nih.gov/, PRJNA1428539.

## Footnotes

1
https://www.cloudtutu.com

2
https://www.bioincloud
